# Correction: Afonso et al. Community-for-Care: An Integrated Response to Informal Post-Caregivers. *Healthcare* 2025, *13*, 3318

**DOI:** 10.3390/healthcare14050686

**Published:** 2026-03-09

**Authors:** Catarina Inês Costa Afonso, Ana Spínola Madeira, Alcinda Reis, Susana Magalhães

**Affiliations:** 1 RISE-Health, Santarém School of Health, Polytechnic Institute of Santarém, Quinta do Mergulhão Srª da Guia, 2005-075 Santarém, Portugal; ana.madeira@essaude.ipsantarem.pt (A.S.M.); alcinda.reis@essaude.ipsantarem.pt (A.R.); 2 i3S—Instituto de Investigação e Inovação em Saúde, Universidade do Porto, 4200-135 Porto, Portugal; susana.magalhaes@i3s.up.pt

## Affiliation Correction

In the original publication [1], there was an error regarding the affiliation for Susana Magalhães. The updated affiliation should be as follows: i3S—Instituto de Investigação e Inovação em Saúde, Universidade do Porto, 4200-135 Porto, Portugal. The authors state that the scientific conclusions are unaffected. This correction was approved by the Academic Editor. The original publication has also been updated.
