# Supplementary material for: Community-for-Care: An Integrated Response to Informal Post-Caregivers
Source: Healthcare (Basel). 2025 Dec 18;13(24):3318. doi: 10.3390/healthcare13243318 (PMC12733144; doi:10.3390/healthcare13243318)
Supplement: Supplementary file 1 [file healthcare-13-03318-s001.zip › healthcare-4007934-supplementary.pdf]

Table S1. Focus Group Question Guide (adapted from Krueger & Casey, 2015 [32])

**Understanding an Issue or Problem**<br>Learning about participants' perceptions of a network of resources to support the informal post-caregivers, focusing on the words they use and their general disposition toward the topic.		
	**Questions**	**Time**
1	When you hear the word caregiving, what comes to your mind?	5 min
2	What do you hear people saying about the network of resources to support the informal post-caregiver?	5 min
3	On a scale of 1 to 5, how concerned are you about this topic? The range is: 1 = not at all concerned to 5 = very concerned.<br>Please give me your number and your reason.	10 min
4	How widespread is a network of resources to support the informal post-caregiver?	10 min
5	Should action be taken on network of resources to support the informal post-caregiver? If yes, then who should take action?	10 min
6	What, if anything, might you do about this problem?	10 min
7	What advice do you have for leaders in your community on this problem?	10 min
	Total	60 min
